# Supplementary material for: Causal Relationship between Aging and Anorexia Nervosa: A White-Matter-Microstructure-Mediated Mendelian Randomization Analysis
Source: Biomedicines. 2024 Aug 16;12(8):1874. doi: 10.3390/biomedicines12081874 (PMC11351342; doi:10.3390/biomedicines12081874)
Supplement: Supplementary file 1 [file biomedicines-12-01874-s001.zip › Supplementary Figure/Figure S8/Leaveoneout plot of MR analysis for TL to ubm-b-1975.pdf]

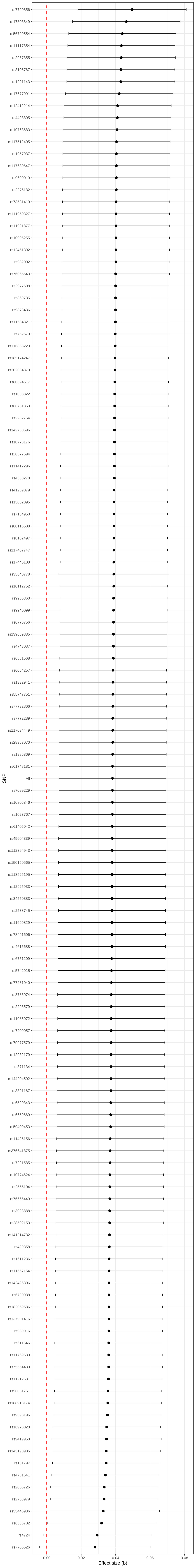
rs7790856
rs17803849
rs56799554
rs11117354
rs2967355
rs8105767
rs1291143
rs17677991
rs12412214
rs4498805
rs10768683
rs117512405
rs1957937
rs117630647
rs9600019
rs2276182
rs73581419
rs111950327
rs11991877
rs10905255
rs12451892
rs932002
rs76065543
rs2977608
rs869785
rs9878436
rs11584821
rs762679
rs116863223
rs185174247
rs202034370
rs80324517
rs1003322
rs66731853
rs2282764
rs142730696
rs10773176
rs28577594
rs11412296
rs4530278
rs41269079
rs13062095
rs7164950
rs80116508
rs8102497
rs117407747
rs17445108
rs35640778
rs10112752
rs9955360
rs9940099
rs6776756
rs139669835
rs4743037
rs6881568
rs6054257
rs1332941
rs55747751
rs77732866
rs7772289
rs117034449
rs28363070
rs1985369
rs61748181
All
rs7099229
rs10805346
rs1023767
rs61405042
rs45604339
rs112394943
rs150150565
rs113525195
rs12925933
rs34550383
rs2538745
rs11699829
rs78491606
rs4616688
rs6751209
rs5742915
rs77231040
rs3785074
rs2293579
rs11085072
rs7209057
rs79977579
rs12932179
rs871134
rs144204502
rs3891167
rs6590343
rs6659669
rs59409453
rs11426156
rs376641875
rs7221585
rs10774624
rs2555104
rs76666449
rs3093888
rs28502153
rs141214782
rs429358
rs1611236
rs11557154
rs142426306
rs6790988
rs182059586
rs137901416
rs939916
rs611646
rs11769630
rs75664430
rs11212631
rs56061761
rs188918174
rs9398196
rs16978028
rs9419958
rs143190905
rs131797
rs4731541
rs2056726
rs2763979
rs35446936
rs6536702
rs4724
rs7705526
SNP
0.00
0.02
0.04
0.06
0.08
Effect size (b)
